# Tendências Temporais na Epidemiologia da Febre Reumática Aguda: Uma Análise Nacional de 2008 a 2022

**DOI:** 10.36660/abc.20240763

**Published:** 2025-07-29

**Authors:** Antonio Mutarelli, Larissa Armelin, Alexandre Negrão Pantaleão, Alleh Nogueira, Carla Jorge Machado, José Luiz P Silva, Jagdip Kang, Walderez O. Dutra, Maria C. P. Nunes

**Affiliations:** 1 Faculdade de Medicina Universidade Federal de Minas Gerais Belo Horizonte MG Brasil Faculdade de Medicina da Universidade Federal de Minas Gerais, Belo Horizonte, MG – Brasil; 2 Cardiac Ultrasound Lab Massachusetts General Hospital Harvard Medical School Boston Massachusetts EUA Cardiac Ultrasound Lab, Massachusetts General Hospital, Harvard Medical School, Boston, Massachusetts – EUA; 3 Escola Bahiana de Medicina e Saúde Pública Salvador BA Brasil Escola Bahiana de Medicina e Saúde Pública, Salvador, BA – Brasil; 4 Departamento de Estatística Universidade Federal do Paraná Curitiba PR Brasil Departamento de Estatística, Universidade Federal do Paraná,Curitiba, PR – Brasil; 5 Departamento de Morfologia Instituto de Ciências Biológicas Universidade Federal de Minas Gerais Belo Horizonte MG Brasil Departamento de Morfologia, Instituto de Ciências Biológicas, Universidade Federal de Minas Gerais, Belo Horizonte, MG – Brasil

**Keywords:** Febre Reumática Aguda, Internação, Prevalência, Brasil, Epidemiologia

## Abstract

**Fundamento:**

A febre reumática aguda (FRA) ainda representa um grande desafio de saúde pública, principalmente em países de baixa e média renda. Afeta desproporcionalmente populações não brancas em regiões menos favorecidas e pode evoluir para cardiopatia reumática (CR), associada à alta morbidade e mortalidade.

**Objetivos:**

Analisar internações e óbitos relacionados à FRA no Brasil entre 2008 e 2022, destacando desigualdades regionais e demográficas.

**Métodos:**

Estudo transversal baseado em dados de internação e mortalidade por FRA, coletados pelo Sistema de Informações Hospitalares (SIH/SUS). Dados estratificados por características demográficas, região e tipo de visita hospitalar foram analisados utilizando modelos de regressão linear de média móvel para avaliar o impacto de idade, sexo e raça. A significância estatística foi estabelecida em p < 0,05.

**Resultados:**

Foram registradas 11.061 internações e 65 óbitos por FRA; 53% dos hospitalizados eram homens e 16% eram brancos. A faixa etária de 10 a 14 anos apresentou as maiores taxas de internação, enquanto a de 15 a 19 anos apresentou mais óbitos. As internações foram mais frequentes entre indivíduos não-brancos, concentrando-se no Nordeste do Brasil. Ao longo do tempo, as internações pela FRA diminuíram em todas as demografias, com convergência gradual entre as taxas de homens e mulheres até 2022.

**Conclusões:**

O estudo revela um declínio nas internações por FRA em todas as regiões e demografias, embora ainda persistam disparidades. Não houve diferença significativa nos casos entre homens e mulheres. Evidência é uma evidência entre fatores socioeconômicos e a carga de doença, afetando mais grupos de baixa renda.

## Graphical abstract

**Figura Central:**
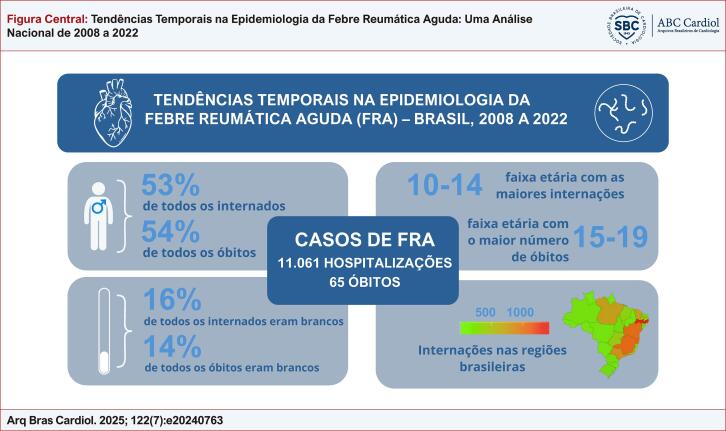
Tendências Temporais na Epidemiologia da Febre Reumática Aguda: Uma Análise Nacional de 2008 a 2022

## Introdução

A febre reumática aguda (FRA) é uma reação inflamatória anormal a uma infecção por estreptococos do grupo A durante a infância ou adolescência e tem como consequência mais grave a doença reumática cardíaca (DCR), uma doença cardíaca valvar.^1^ Os critérios diagnósticos de Jones descrevem a apresentação mais comum: envolvimento articular, distúrbio valvar cardíaco, nódulos subcutâneos, erupção cutânea e coreia de Sydenham.^2^ Entretanto, o envolvimento cardíaco e valvar é a principal causa da carga da doença, que pode levar à internação, morte e DCR crônica.^3^

DCR afeta mais de 40 milhões de indivíduos e resulta em mais de 300 mil mortes anualmente.^4^ Após um episódio de FRA, a lesão valvar progride após episódios clínicos recorrentes claramente evidentes ou infecções estreptocócicas subclínicas e pode ser diagnosticada por meio de triagem sistemática ecocardiográfica ou após o surgimento dos sintomas.^1^ A terapia antibiótica adequada para infecções de garganta pode prevenir a FRA, e a profilaxia secundária após um episódio de FRA pode ajudar a prevenir o desenvolvimento de DCR.^5^ Além disso, a profilaxia secundária com penicilina para DCR subclínica (detectada por ecocardiograma) reduz o risco de progressão da doença.^6^

A prevalência de DCR aumentou em mais de 70% nos últimos 30 anos e apresenta uma prevalência três vezes maior em mulheres.^7,8^ As razões para essa diferença entre os sexos ainda são pouco compreendidas. Um estudo sugere que a protimosina-alfa, uma proteína altamente expressa na DCR e associada aos receptores de estrogênio, modula as respostas imunológicas. Essa interação pode aumentar o reconhecimento de epítopos mimetizadores de colágeno tipo 1 por células T CD8+ na DCR, contribuindo potencialmente para a ativação autoimune.^9^

A FRA continua sendo um desafio significativo para a saúde pública, especialmente em países de baixa e média renda, onde suas consequências de longo prazo, a DCR, levam a morbidade e mortalidade substanciais.^10^ Apesar dos esforços para controlar e prevenir a FRA por meio de melhor acesso à assistência médica e profilaxia com antibióticos, as disparidades persistem tanto na prevalência quanto nos resultados da doença entre diferentes populações.^11^

Entender as tendências epidemiológicas e as disparidades demográficas é essencial para refinar estratégias de prevenção e otimizar a alocação de recursos de saúde. No entanto, ainda há uma falta de dados contemporâneos sobre a carga da FRA na era atual de profilaxia primária e secundária.Para abordar a lacuna da literatura sobre gravidade e mortalidade por FRA, conduzimos um estudo epidemiológico utilizando o Sistema de Informações Hospitalares (SIH) do Sistema Único de Saúde (SUS).^12^ O SIH coleta seus dados por meio da Autorização de Internação Hospitalar (AIH), utilizada por hospitais públicos e privados conveniados ao SUS.^13^ As AIHs são documentos preenchidos para cada paciente, permitindo a coleta de mais de 50 variáveis, incluindo o motivo da internação, com diagnósticos codificados de acordo com a CID-10. As unidades hospitalares enviam esses documentos aos gestores municipais ou estaduais, que consolidam as informações e as encaminham a um departamento do Ministério da Saúde. Este departamento então processa os dados no DATASUS e gera créditos para os procedimentos registrados nas AIHs. Nosso objetivo foi analisar as tendências anuais da prevalência de FRA no Brasil, fornecendo insights sobre a evolução da carga da doença e o impacto dos esforços de prevenção.

## Métodos

### Desenho do estudo

Realizamos um estudo ecológico transversal utilizando dados de internação e mortalidade por FRA registrados no SIH do Ministério da Saúde. O SIH é um banco de dados secundário disponível no Departamento de Informática do Sistema Único de Saúde (DATASUS).^14^ Os dados são apresentados pelo TABNET, uma ferramenta de tabulação desenvolvida pelo DATASUS.^14^ Esta ferramenta estratifica dados de internação por características demográficas, geográficas, de custo e outros fatores relevantes.^14^ Dados de internação hospitalar abrangendo os anos de 2008 a 2022 foram coletados para comparar as taxas de internação e mortalidade entre homens e mulheres com FRA. Como parte das análises secundárias, avaliamos o número de internações e mortalidade por região geográfica (Norte, Nordeste, Centro-Oeste, Sudeste e Sul), faixa etária (5 a 9, 10 a 14 e 15 a 19 anos), raça/etnia autodeclarada (branca, não branca e desconhecida) e tipo de consulta médica (eletiva ou urgente). Vale ressaltar que o DATASUS está disponível publicamente para pacientes anonimizados e não requer aprovação de comitês de ética.^15^

### Coleta de dados

Dois autores independentes (AM e LA) coletaram os dados; não foram encontradas discrepâncias entre seus conjuntos de dados. Os dados foram coletados do DATASUS, a plataforma de coleta de dados do sistema público de saúde brasileiro. O DATASUS foi criado em 1991. O acesso aos dados de produção hospitalar na plataforma está disponível de 1992 a 2007 e de 2008 em diante.^14^ Essa fragmentação possivelmente se deve à unificação da Tabela de Procedimentos em 2008, que trouxe mudanças significativas às AIHs.^16^ O ano de 2023 foi excluído deste estudo devido à indisponibilidade de dados completos no momento da coleta. Até onde sabemos, nenhuma análise com o mesmo objetivo de pesquisa foi conduzida com dados do DATASUS. O período escolhido, de 2008 a 2022, foi selecionado para manter a consistência nos métodos de coleta de dados. Excluímos pacientes com menos de cinco anos e mais de 19 anos para minimizar possíveis vieses, com foco na faixa etária em que a IRA é mais prevalente.^17^

Coletamos dados sobre as taxas de internação e mortalidade relacionadas à FRA, com foco específico em indivíduos de 5 a 19 anos. Filtramos os dados por ano, sexo, raça, tipo de consulta médica (eletiva ou urgente) e região do país. Além disso, para comparação do tamanho da amostra, obtivemos o número total de homens e mulheres para cada ano de 2008 a 2022, residentes no Brasil.

### Análise estatística

As frequências de internações foram estratificadas por idade, sexo e raça/etnia. As tendências temporais foram representadas por gráficos de linhas. Mapas foram utilizados para mostrar a heterogeneidade regional dentro e ao longo dos anos. Modelos de média móvel autorregressiva linear generalizada (GLARMA) foram utilizados para explorar o efeito da idade, sexo e raça nas séries temporais.^18^ Essa classe de modelos permite fazer inferências sobre variáveis de regressão, ao mesmo tempo em que considera adequadamente a dependência serial de séries temporais discretas. Distribuições binomial e binomial negativa foram consideradas para as proporções e números de internações, respectivamente. Termos de interação entre os fatores foram incluídos nos modelos. A adequação dos modelos foi avaliada pela inspeção dos gráficos de resíduos preditivos. Um teste qui-quadrado foi realizado para avaliar as associações entre características demográficas e clínicas e os desfechos de internação ou óbito (Tabela 1). Tabelas de contingência foram construídas para dados de internação e óbito, e valores de p foram calculados para determinar significância estatística. A análise estatística foi realizada com R (versão 4.4.1, Equipe Principal R) usando os pacotes *tidyverse, ggpubr* e *glarma*. A significância estatística foi estabelecida em p < 0,05.

**Tabela 1:** – Epidemiologia e dados demográficos de pacientes com febre reumática aguda no Brasil

	Internação					Morte				
	Masculino		Feminino		Valor p	Masculino		Feminino		Valor p
Total	5.907	(53,4%)	5.154	(46,6%)	0,000	35	(53,8%)	30	(46,2%)	0,535
**Raça**										
Branca	928	(15,7%)	834	(16,2%)	0,025	6	(17,1%)	3	(10,0%)	0,317
Não-branca	2.545	(43,1%)	2.182	(42,3%)	0,000	12	(34,3%)	9	(30,0%)	0,513
Não informada	2.434	(41,2%)	2.138	(41,5%)	0,000	17	(48,6%)	18	(60,0%)	0,866
**Faixa etária**										
5 - 9	2.134	(36,1%)	1.755	(34,1%)	0,000	5	(14,3%)	5	(16,7%)	1.000
10 - 14	2.650	(44,9%)	2.214	(43,0%)	0,000	14	(40,0%)	12	(40,0%)	0,695
15 - 19	1.123	(19,0%)	1.185	(23,0%)	0,197	16	(45,7%)	13	(43,3%)	0,578
**Natureza da consulta médica**										
Eletiva	620	(10,5%)	588	(11,4%)	0,357	4	(11,4%)	2	(6,7%)	0,414
Urgência	5.287	(89,5%)	4.566	(88,6%)	0,000	31	(88,6%)	28	(93,3%)	0,696
**Intervalo de anos**										
2008-2012	3.282	(55,6%)	2.684	(52,1%)	0,000	15	(42,9%)	21	(70,0%)	0,317
2013-2017	1.675	(28,4%)	1.552	(30,1%)	0,030	13	(37,1%)	7	(23,3%)	0,180
2018-2022	950	(16,1%)	918	(17,8%)	0,460	7	(20,0%)	2	(6,7%)	0,096

## Resultados

Nossa busca no DATASUS revelou um total de 11.061 internações e 65 mortes por FRA. Entre os pacientes hospitalizados, 53% eram do sexo masculino e 16% auto-identificadas como brancas (Tabela 1). A faixa etária associada às maiores internações foi a de 10 a 14 anos, enquanto o maior número de óbitos ocorreu na faixa etária de 15 a 19 anos, totalizando 29 mortes. Notavelmente, a grande maioria (89%) das consultas médicas foram classificadas como urgentes.A região do Nordeste Brasileiro apresentou as maiores frequências de internação por FRA(44%). Detalhes adicionais podem ser encontrados na Tabela 1, e um resumo do nosso estudo pode ser encontrado na Figura Central.

**Figura 1:**
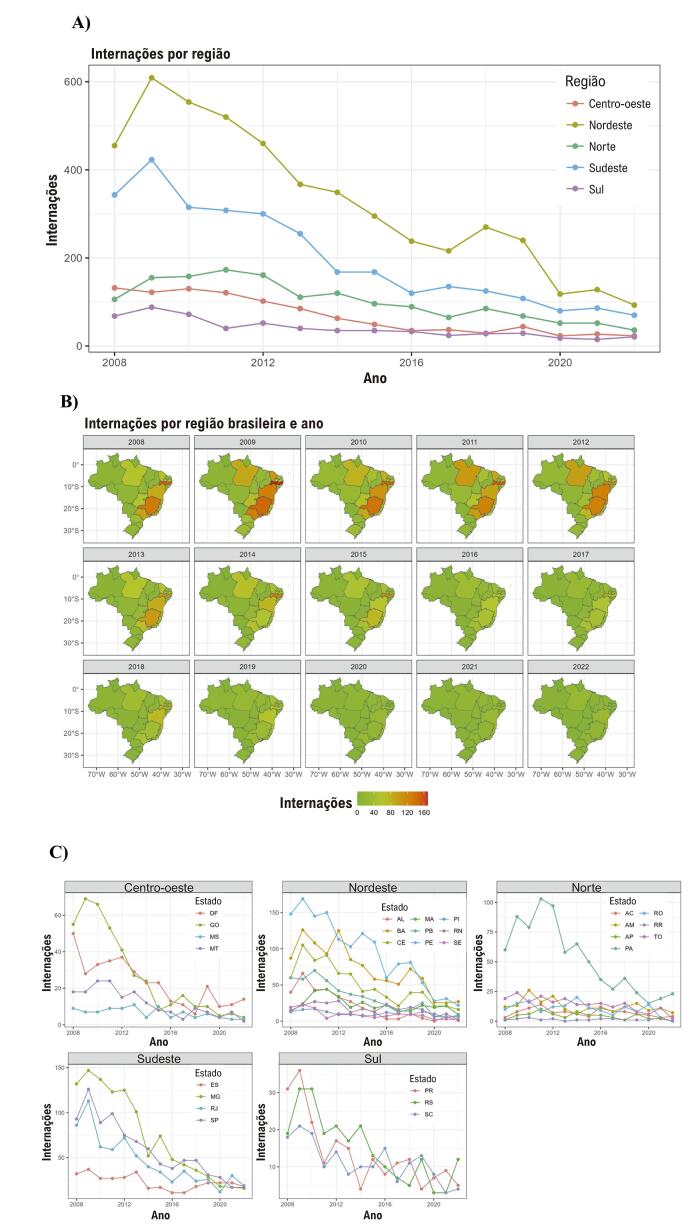
– Internação por febre reumática aguda por ano e região. A) Internação por ano e região: gráfico linear. B) Internação por ano e estados: mapa dos estados brasileiros. C) Internação por ano, região e estado: gráfico linear.

**Figura 2:**
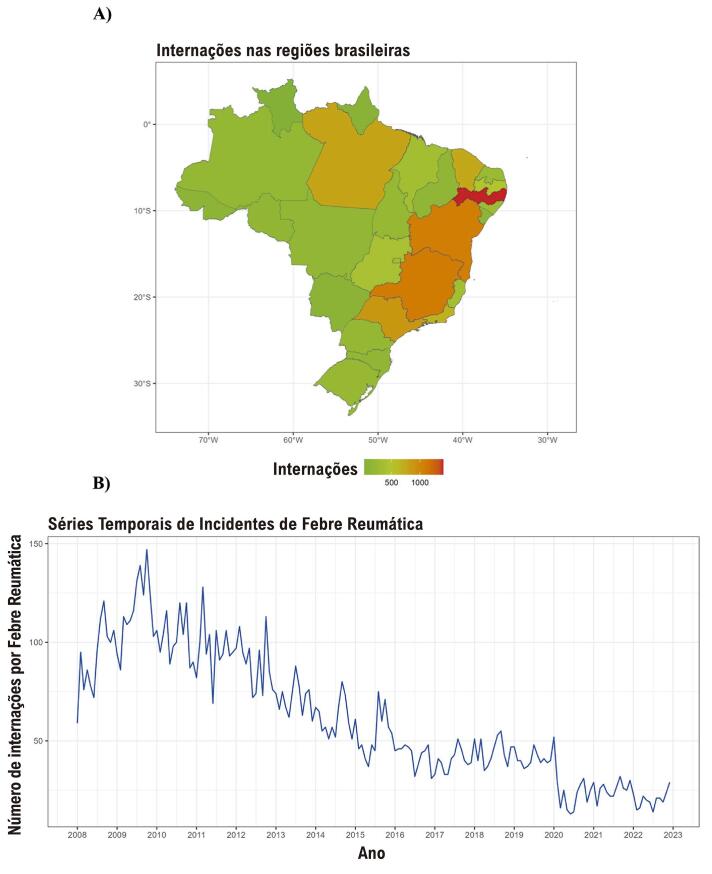
– Internação por febre reumática aguda. A) Internação por estado brasileiro (2008-2022): mapa dos estados brasileiros. B) Internação por ano: gráfico linear sazonal.

As internações por FRA apresentaram uma redução consistente em todos os grupos demográficos e regiões (Figura 1). Notavelmente, as regiões Nordeste e Sudeste foram associadas às maiores frequências de internação por FRA (Figura 1). Por outro lado, a região Sul apresentou menos casos de FRA, com taxas de frequência permanecendo relativamente estáveis ao longo do tempo. Particularmente notáveis são os estados de Pernambuco, Bahia, Minas Gerais e São Paulo, que apresentaram taxas de internação por FRA consistentemente altas durante o período do estudo, formando um corredor terrestre contíguo de casos elevados de FRA (Figura 2a). Há uma tendência sazonal de aumento da internação por FRA próximo aos meses de inverno no hemisfério sul (Figura 2b).

**Figura 3:**
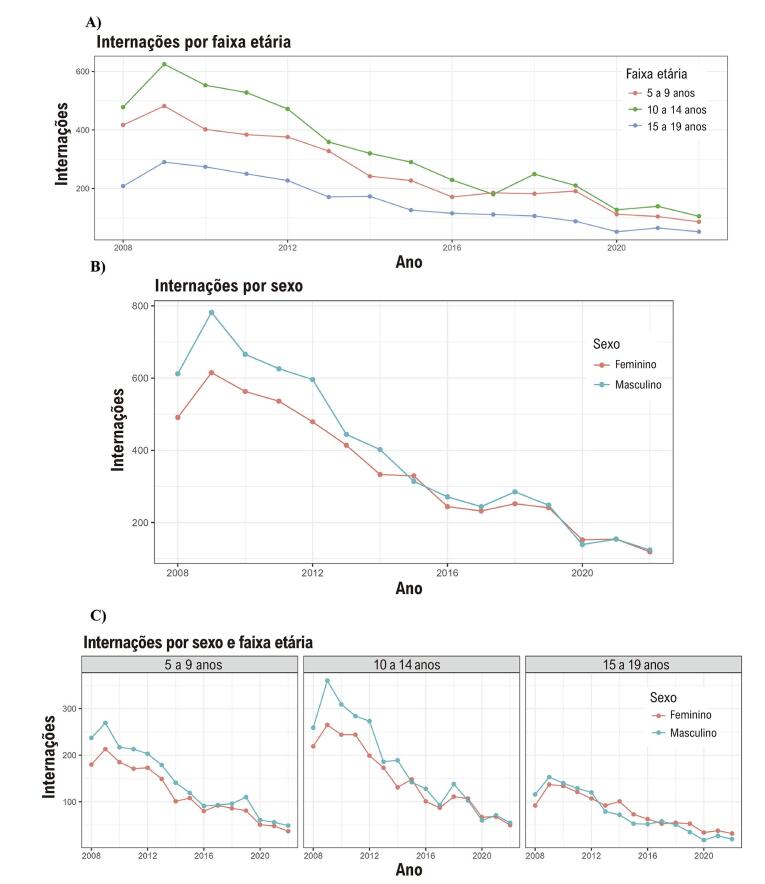
– Internação por febre reumática aguda. A) Internação por faixa etária: gráfico linear. B) Internação por sexo: gráfico linear. C) Internação por sexo e faixa etária: gráfico linear.

Maiores internações foram associadas aos homens no intervalo 2008 - 2012 (Figura 3b). Após esse período, a comparação de internação entre homens e mulheres gradualmente se sobrepôs até a convergência em 2022. Além disso, os casos de FRA ao longo do tempo apresentaram uma disparidade notável entre a faixa etária de 5 a 14 anos em comparação com a faixa etária de 15 a 19 anos, com a diferença diminuindo gradualmente ao longo do tempo (Figura 3a). Especificamente, o número de casos foi maior na faixa etária de 10 a 14 anos em comparação com a faixa etária de 5 a 9 anos, com casos se sobrepondo após 2017. Há também um gráfico de incidência de internação por idade e sexo (Figura Suplementar 1). A análise ao longo do tempo comparando faixas etárias e sexo revelou uma maior frequência de internação por FRA em homens nas faixas etárias de 5 a 9 anos e 10 a 14 anos durante os anos iniciais do estudo (Figura 3c). No entanto, na faixa etária de 15 a 19 anos, os números de internação por FRA representando homens e mulheres se sobrepõem consistentemente do início ao fim do período do estudo.

**Figura 4:**
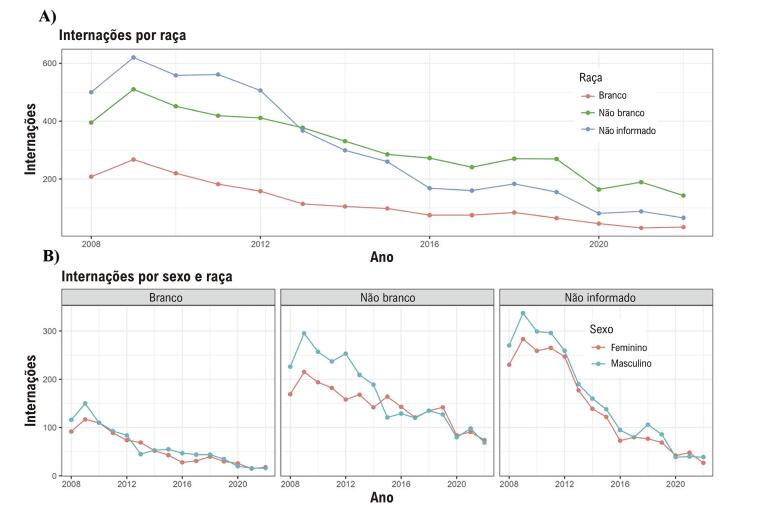
– Internação por febre reumática aguda. A) Internação por raça: gráfico linear. B) Internação por raça e sexo: gráfico linear.

Da mesma forma, ao examinar sexo e raça, o número de casos de indivíduos brancos se sobrepõe ao longo de todo o período do estudo (Figura 4a). Entre indivíduos não brancos, a internação masculina é inicialmente maior, alinhando-se gradualmente à internação feminina ao longo do tempo. As internações entre indivíduos não brancos superaram consistentemente as internações entre indivíduos brancos, mas a magnitude dessa diferença diminuiu gradualmente ao longo do tempo (Figura 4).

## Discussão

Em um estudo ecológico transversal, observamos que, entre as 11.061 internações por FRA na última década, 53% eram do sexo masculino. A taxa de internação foi significativamente maior entre homens de 5 a 14 anos. Além disso, ao longo do período do estudo, as internações por FRA apresentaram uma redução consistente em todos os grupos demográficos e regiões.

No presente estudo, o número de internações em homens foi maior do que em mulheres, totalizando 53% entre os 11.061 analisados. Alguns estudos mencionam que a prevalência de FRA não difere significativamente entre os sexos na maioria das populações.^1^ sem evidências concretas para apoiar esse argumento. Negi et al. incluíram prospectivamente 2.475 pacientes com FRA ou DCR e revelaram que a preponderância feminina surge apenas após os 20 anos de idade.^19^ No entanto, apenas 15 pacientes com FRA foram incluídos nesse estudo. Lawrence et al. analisaram 615 casos de FRA no norte da Austrália e revelaram que as mulheres tinham 1,5 vez mais probabilidade de apresentar FRA.^20^ Nosso estudo incluiu apenas pacientes hospitalizados por FRA. No entanto, os homens representaram 53% dos casos. Os resultados do presente estudo sugerem que pode haver uma diferença substancial na predisposição sexual entre as fases aguda, subclínica e crônica da doença. Notavelmente, se observa uma tendência de aumento da predominância feminina à medida que a doença progride.^19-21^ Para uma compreensão mais clara da mudança na prevalência entre mulheres com FRA e DCR, pacientes com FRA devem ser acompanhadas ao longo do tempo para analisar a predisposição sexual na progressão da doença.

Além disso, demonstramos que a diferença nos casos de FRA entre os sexos foi maior no subgrupo de 5 a 9 anos, com convergência entre as internações por FRA por gênero nos subgrupos de 10 a 14 anos e 15 a 19 anos. O aumento da prevalência de CR em mulheres relacionado à idade foi relatado em outros estudos.^8,20-24^ Uma explicação plausível para tais evidências é a maior suscetibilidade autoimune em mulheres devido aos efeitos do estrogênio.^9^ Foi demonstrado que a protimosina alfa, que está associada aos receptores de estradiol, está implicada na citotoxicidade das células T CD8+ contra o colágeno tipo 1 (sugerindo mecanismos que provocam autoimunidade) e pode contribuir para a predisposição feminina na DCR.^9^

Em relação à etnia, as internações entre indivíduos não brancos superaram consistentemente as de brancos, com a região Nordeste apresentando um número maior de casos de FRA do que outras regiões brasileiras. As diferenças no risco de FRA entre populações ao redor do mundo são explicadas principalmente por fatores ambientais, sendo a associação entre FRA e pobreza e desvantagem econômica bem estabelecida.^25^ Nesse ponto, a superlotação domiciliar é o fator de risco mais bem descrito, cuja resolução está associada à diminuição da prevalência de FRA em países desenvolvidos ao longo do século XX.^26^ Além disso, melhorias nos cuidados médicos e na educação em saúde também estão associadas a uma menor prevalência de FRA.^27,28^ Infelizmente, o Brasil ainda apresenta grande desigualdade racial e regional devido a fatores históricos. Nesse sentido, a população não branca e a região Nordeste costumam apresentar piores condições socioeconômicas, o que pode explicar os resultados.^29,30^

As internações devido à FRA diminuíram consistentemente em todos os grupos demográficos e regiões, apesar da crescente prevalência de DCR nos últimos 30 anos.^7^ O declínio global na prevalência de FRA é atribuído a medidas de saúde pública, especialmente à profilaxia antibiótica e à melhoria do saneamento básico. No entanto, a prevalência de DCR permanece significativa devido a infecções estreptocócicas mal tratadas e episódios de FRA de décadas atrás. O aumento da prevalência de DCR também está associado à maior expectativa de vida dos pacientes, impulsionada por melhores tratamentos médicos, ao aumento da valvoplastia mitral percutânea por balão e à profilaxia com penicilina para retardar a progressão da doença.

Nosso artigo apresenta algumas limitações. Primeiramente, o subconjunto de pacientes incluído nesta análise pode não ser representativo de todos os pacientes com FRA, visto que avaliamos apenas as internações por FRA. Além disso, não é possível atestar a qualidade dos prontuários médicos que embasam os dados no banco de dados utilizado. Este é um estudo ecológico, e as estimativas não foram ajustadas para fatores influentes, como fatores socioeconômicos. A descoberta da diferença entre os sexos se refere apenas à internação, e não podemos fornecer justificativas metodológicas para isso.

## Conclusão

Esta análise nacionalmente representativa dos registros de internações da população brasileira de 2008 a 2022 demonstra uma clara redução nas internações por FRA ao longo do tempo. Além disso, não há diferença significativa nos casos de FRA entre homens e mulheres. O estudo destaca uma correlação entre fatores socioeconômicos e a carga da doença, com grupos de baixa renda apresentando uma taxa maior de internações por FRA.
