# Supplementary material for: Tendências Temporais na Epidemiologia da Febre Reumática Aguda: Uma Análise Nacional de 2008 a 2022
Source: Arq Bras Cardiol. 2025 Jul 29;122(7):e20240763. [Article in Portuguese] doi: 10.36660/abc.20240763 (PMC12695096; doi:10.36660/abc.20240763)
Supplement: Translation

# Translation: Temporal Trends in the Epidemiology of Acute Rheumatic Fever: A Nationwide Analysis from 2008 to 2022

**DOI:** 10.36660/abc.20240763i

**Authors:** Antonio Mutarelli, Larissa Armelin, Alexandre Negrão Pantaleão, Alleh Nogueira, Carla Jorge Machado, José Luiz P Silva, Jagdip Kang, Walderez O. Dutra, Maria C. P. Nunes

**Affiliations:** 1 Brazil Faculdade de Medicina da Universidade Federal de Minas Gerais, Belo Horizonte, MG – Brazil; 2 USA Cardiac Ultrasound Lab, Massachusetts General Hospital, Harvard Medical School, Boston, Massachusetts – USA; 3 Brazil Escola Bahiana de Medicina e Saúde Pública, Salvador, BA – Brazil; 4 Brazil Departamento de Estatística, Universidade Federal do Paraná, Curitiba, PR – Brazil; 5 Brazil Departamento de Morfologia, Instituto de Ciências Biológicas, Universidade Federal de Minas Gerais, Belo Horizonte, MG – Brazil

**Keywords:** Acute Rheumatic Fever, Hospitalization, Prevalence, Brazil, Epidemiology

## Abstract

**Background:**

Acute rheumatic fever (ARF) remains a significant public health challenge, especially in low- and middle-income countries. It disproportionately affects non-white populations in underprivileged regions and may lead to rheumatic heart disease (RHD), which has high morbidity and mortality.

**Objectives:**

Analyze hospitalizations and deaths related to ARF in Brazil between 2008 and 2022, highlighting regional and demographic inequalities.

**Methods:**

We conducted a cross-sectional study analyzing hospitalization and mortality data caused by ARF from 2008 to 2022 in Brazil, collected through the Hospital Information System (SIH/SUS). Data were stratified by demographics, region, and hospital visit type (urgent or elective) and analyzed using generalized linear autoregressive moving average models to assess the impact of age, sex, and race. Statistical significance was set at p < 0.05.

**Results:**

Of 11,061 hospitalizations and 65 deaths from ARF, 53% were male and 16% were white. The 10–14-year-old age group had the highest hospitalization rates, while the 15–19-year-old group had more deaths. Hospitalizations were higher among non-white individuals and concentrated in Brazil’s Northeast. Over time, ARF-related hospitalizations declined across all demographics, with a gradual convergence between male and female rates by 2022.

**Conclusion:**

Our findings highlight a decline in ARF-related hospitalizations across regions and demographics, though disparities remain. There is no significant difference in ARF cases between men and women. The study highlights a correlation between socioeconomic factors and disease burden, with low-income groups experiencing a higher rate of ARF hospitalizations.

## Introduction

Acute rheumatic fever (ARF) is an abnormal inflammatory reaction to a group A streptococcal infection during childhood or adolescence and has its most severe consequence, rheumatic heart disease (RHD), a valvular heart disease.^1^ The Jones diagnostic criteria outline the most common presentation, joint involvement, cardiac valvular disorder, subcutaneous nodules, rash, and Sydenham’s chorea.^2^ However, cardiac and valve involvement is the leading cause of the burden of the disease to hospitalization, death, and chronic RHD.^3^

RHD affects over 40 million individuals and results in more than 300 thousand deaths annually.^4^ Following an ARF episode, the valvular injury progresses after recurrent clinically evident or subclinical streptococcal infections and may be diagnosed through echocardiographic systematic screening or after symptoms arise.^1^ Adequate antibiotic therapy for throat infections can prevent ARF, and secondary prophylaxis after an ARF episode can help prevent the development of RHD.^5^ Furthermore, secondary prophylaxis with penicillin for subclinical (echocardiogram-detected) RHD reduces the risk of disease progression.^6^

RHD prevalence increased by more than 70% in the last 30 years and exhibits a threefold higher prevalence in women.^7,8^ The reasons for this sex difference remain poorly understood. A study suggests that prothymosin-alpha, a protein highly expressed in RHD and associated with estrogen receptors, modulates immune responses. This interaction may enhance CD8+ T-cell recognition of type 1 collagen mimic epitopes in RHD, potentially contributing to autoimmune activation.^9^

ARF remains a significant public health challenge, particularly in low- and middle-income countries, where its long-term consequences, RHD, lead to substantial morbidity and mortality.^10^ Despite efforts to manage and prevent ARF through improved access to healthcare and antibiotic prophylaxis, disparities persist in both the prevalence and outcomes of the disease across different populations.^11^

Understanding the epidemiological trends and demographic disparities is essential for refining prevention strategies and optimizing healthcare resource allocation. However, there remains a lack of contemporary data on the burden of ARF in the current era of primary and secondary prophylaxis. To address the literature gap on ARF severity and mortality, we conducted an epidemiological study utilizing the Hospital Information System (SIH) of the Brazilian National Health System (SUS).^12^ The SIH collects its data through Hospital Admission Authorization (AIH), used by public and private hospitals affiliated with the SUS. ^13^ The AIHs are documents completed for each patient, allowing the collection of more than 50 variables, including the reason for hospitalization, with diagnoses coded according to the ICD-10. Hospital units send these documents to municipal or state managers, who consolidate the information and forward it to a department of the Ministry of Health. This department then processes the data in DATASUS and generates credits for the procedures recorded in the AIHs. We aimed to analyze the annual trends in ARF prevalence in Brazil, providing insights into the evolving burden of the disease and the impact of prevention efforts.

## Methods

### Study design

We conducted a cross-sectional ecological study using inpatient and mortality data from ARF recorded in the SIH of the Ministry of Health of Brazil. SIH is a secondary database available at the Informatics Department of the Brazilian National Health System (DATASUS).^14^ The data is presented by TABNET, a tabulation tool developed by DATASUS.^14^ This tool stratifies inpatient data by demographic, geographic, cost characteristics, and other relevant factors.^14^ Inpatient data spanning the years 2008 to 2022 were collected to compare hospitalization and mortality rates between males and females with ARF. As part of secondary analyses, we assessed the number of hospitalizations and mortality by geographic region (North, Northeast, Midwest, Southeast, and South), age group (5-9, 10-14, and 15-19 years old), race/self-declared ethnicity (white, non-white and unknown), and type of medical visit (elective or urgent). It should be noted that DATASUS is publicly available with de-identified patients and does not require approval from ethics committees.^15^

### Data collection

Two independent authors (AM and LA) collected the data; no discrepancies were found between their datasets. Data were gathered from the DATASUS, the Brazilian public health system’s data collection platform. DATASUS was created in 1991. Access to hospital production data on the platform is available from 1992 to 2007 and from 2008 onwards.^14^ This fragmentation is possibly due to the unification of the Table of Procedures in 2008, which brought significant changes to the AIHs.^16^ The year 2023 was excluded from this study due to the unavailability of complete data at the time of collection. To our knowledge, no analysis with the same research objective has been conducted with DATASUS data. The chosen time frame, from 2008 to 2022, was selected to maintain consistency in data collection methods. We excluded patients aged below five and above 19 years to minimize potential bias, focusing on the age range where ARF is more prevalent.^17^

We collected data on hospitalization and mortality rates related to ARF, specifically focusing on individuals aged 5 to 19 years old. We filtered the data by year, sex, race, the type of medical visit (elective or urgent), and the country region. Furthermore, for sample size comparison, we obtained the total number of males and females for each year from 2008 to 2022, residing in Brazil.

### Statistical analysis

Frequencies of hospitalizations were stratified by age, sex, and race/ethnicity. Temporal trends were represented by line plots. Maps were used to show regional heterogeneity within and over the years. Generalized linear autoregressive moving average (GLARMA) models were used to explore the effect of age, sex, and race on the time series.^18^ This class of models allows making inferences about regression variables while properly accounting for the serial dependence of discrete time series. Binomial and negative binomial distributions were considered for the proportions and numbers of hospitalizations, respectively. Interaction terms between the factors were included in the models. The adequacy of the models was assessed by inspection of plots of predictive residuals. A chi-square test was performed to assess associations between demographic and clinical characteristics and the outcomes of hospitalization or death (Table 1). Contingency tables were constructed for hospitalization and death data, and p-values were calculated to determine statistical significance. Statistical analysis was performed with R (version 4.4.1, R Core Team) using the *tidyverse*, *ggpubr*, and *glarma* packages. Statistical significance was set at p < 0.05.

**Table 1:** – Epidemiology and demographic data from acute rheumatic fever patients in Brazil

	Hospitalization					Death				
	Male		Female		p value	Male		Female		p value
Total	5.907	(53.4%)	5.154	(46.6%)	0.000	35	(53.8%)	30	(46.2%)	0.535
**Race**										
White	928	(15.7%)	834	(16.2%)	0.025	6	(17.1%)	3	(10.0%)	0.317
Non-White	2.545	(43.1%)	2.182	(42.3%)	0.000	12	(34.3%)	9	(30.0%)	0.513
Not informed	2.434	(41.2%)	2.138	(41.5%)	0.000	17	(48.6%)	18	(60.0%)	0.866
**Age group**										
5 - 9	2.134	(36.1%)	1.755	(34.1%)	0.000	5	(14.3%)	5	(16.7%)	1.000
10 - 14	2.650	(44.9%)	2.214	(43.0%)	0.000	14	(40.0%)	12	(40.0%)	0.695
15 - 19	1.123	(19.0%)	1.185	(23.0%)	0.197	16	(45.7%)	13	(43.3%)	0.578
**Nature of medical visit**										
Eletive	620	(10.5%)	588	(11.4%)	0.357	4	(11.4%)	2	(6.7%)	0.414
Urgency	5.287	(89.5%)	4.566	(88.6%)	0.000	31	(88.6%)	28	(93.3%)	0.696
**Year range**										
2008-2012	3.282	(55.6%)	2.684	(52.1%)	0.000	15	(42.9%)	21	(70.0%)	0.317
2013-2017	1.675	(28.4%)	1.552	(30.1%)	0.030	13	(37.1%)	7	(23.3%)	0.180
2018-2022	950	(16.1%)	918	(17.8%)	0.460	7	(20.0%)	2	(6.7%)	0.096

## Results

Our search in the DATASUS revealed a total of 11,061 hospitalizations and 65 deaths from ARF. Among the hospitalized patients, 53% were male, and 16% self-identified as white (Table 1). The age group associated with the highest hospitalizations was 10-14 years old, while a higher number of deaths occurred in the 15-19 age range, totaling 29 deaths. Notably, the vast majority (89%) of medical visits were classified as urgent. The Northeastern Brazilian region exhibited the highest frequencies of ARF hospitalization (44%). Additional details can be found in Table 1, and a summary of our study can be found in the Central Illustration.

Hospitalizations due to ARF exhibited a consistent decrease across all demographic groups and regions (Figure. 1). Notably, the Northeast and Southeast regions were associated with the highest frequencies of ARF hospitalization (Figure. 1). Conversely, the South region experienced fewer cases of ARF, with frequency rates remaining relatively steady over time. Particularly noteworthy are the states of Pernambuco, Bahia, Minas Gerais, and São Paulo, which exhibited consistently high-frequency ARF hospitalization rates over the study period, forming a contiguous land corridor of elevated ARF cases (Figure. 2a). There is a seasonal trend of ARF hospitalization increasing near the winter months of the south hemisphere (Figure. 2b).

**Figure 1:** – Acute rheumatic fever hospitalization by year and region. A – Hospitalization by year and region: linear graph. B – Hospitalization by year and states: Brazilian states map. C – Hospitalization by year, region, and state: linear graph.

**Figure 2:** – Acute rheumatic fever hospitalization. A – Hospitalization by Brazilian state (2008-2022): Brazilian states map. B – Hospitalization by year: seasonal linear graph.

Higher hospitalizations were associated with males over the 2008–2012 interval (Figure 3b). After this period, hospitalization comparison between males and females gradually overlapped until convergence by 2022. Additionally, the ARF cases over time displayed a notable disparity between the 5-14 age group compared to the 15-19 age group, with the difference gradually diminishing over time (Figure. 3a). Specifically, the number of cases was higher in the 10-14 age range compared to the 5-9 age group, with cases overlapping after 2017. There is also an incidence graph on hospitalization by age and sex (Supplementary Figure. 1) Analysis over time comparing age ranges and sex revealed a higher ARF hospitalization frequency of males in the age groups of 5-9 and 10-14 during the initial years of the study (Figure. 3c). However, in the age range of 15-19, the ARF hospitalization numbers representing male and female overlap consistently from the beginning to the end of the study period.

**Figure 3:** – Acute rheumatic fever hospitalization. A – Hospitalization by age group: linear graph. B – Hospitalization by sex: linear graph. C – Hospitalization by sex and age group: linear graph.

Similarly, when examining sex and race, the number of cases of white individuals overlaps throughout the entire study period (Figure 4a). Among non-white individuals, male hospitalization is initially greater, gradually aligning with female hospitalization over time. Hospitalizations among non-white individuals consistently outnumbered those among white individuals, but the magnitude of this difference gradually diminished over time (Figure 4).

**Figure 4:** – Acute rheumatic fever hospitalization. A – Hospitalization by race: linear graph. B – Hospitalization by race and sex: linear graph.

## Discussion

In this cross-sectional ecological study, we observed that among the 11,061 ARF hospitalizations over the past decade, 53% were male. The hospitalization rate was significantly higher among males aged 5-14 years. Furthermore, over the study period, hospitalizations due to ARF exhibited a consistent decrease across all demographic groups and regions.

In the present study, the number of hospitalizations in men was higher than in women, totaling 53% among the 11,061 analyzed. Some studies mention that the prevalence of ARF is not significantly different between sexes in most populations,^1^ without concrete evidence to support this argument. Negi et al. prospectively included 2475 patients with ARF or RHD and revealed that the female preponderance arises only after 20 years of age.^19^ However, only 15 patients with ARF were included in that study. Lawrence et al. analyzed 615 cases of ARF in Northern Australia and revealed that females were 1.5 times more likely to present with ARF.^20^ Our study included only patients hospitalized due to ARF. Nevertheless, males represented 53% of the cases. The findings of the present study suggest that there may be a substantial difference in sex predisposition across the acute, subclinical, and chronic phases of the disease. Notably, a trend toward increasing female predominance appears as the disease progresses.^19-21^ For a clearer understanding of the change in prevalence among women with ARF and RHD, patients with ARF should be followed over time to analyze sex predisposition in the progression of the disease.

Moreover, we demonstrated that the difference in ARF cases between sexes was greater in the 5–9-year-old subgroup, with convergence between gender ARF hospitalization in the 10-14 and 15-19 age subgroups. The age-related increase in the female prevalence of RHD has been reported in other studies.^8,20-24^ One plausible explanation for such evidence is greater autoimmune susceptibility in women due to estrogen effects.^9^ It has been demonstrated that prothymosin alpha, which is associated with estradiol receptors, is implicated in CD8+ T-cell cytotoxicity against type 1 collagen (suggesting mechanisms provoking autoimmunity) and may contribute to female predisposition in RHD.^9^

Regarding ethnicity, hospitalizations among non-white individuals consistently outnumbered white individuals, with the Northeastern region exhibiting a higher number of ARF cases than other Brazilian regions. The differences in risk of ARF between populations around the world are mostly explained by environmental factors, with the association between ARF and poverty and economic disadvantage being well established.^25^ On this point, household overcrowding is the best-described risk factor, whose resolution is associated with a decrease in ARF prevalence in developed countries through the twentieth century.^26^ Additionally, improvements in medical care and health education are also associated with a lower prevalence of ARF.^27,28^ Unfortunately, Brazil still has great racial and regional inequality due to historical factors. In this regard, the non-white population and the Northeast region typically maintain worse socioeconomic which may explain the findings.^29,30^

Hospitalizations due to ARF consistently decreased across all demographics and regions despite the rising RHD prevalence in the last 30 years.^7^ The global decline in ARF prevalence is attributed to public health measures, especially antibiotic prophylaxis and improved sanitation. However, RHD prevalence remains significant due to poorly treated streptococcal infections and ARF episodes from decades ago. Increased RHD prevalence is also linked to patients’ longer life expectancy, driven by better medical therapy, the rise of percutaneous balloon mitral valvuloplasty, and penicillin prophylaxis to slow disease progression.

Our paper has some limitations. First, the subset of patients included in this analysis may not be representative of all ARF patients since we evaluated only hospitalizations by ARF. Furthermore, there is an inability to attest to the quality of the medical records that support the data in the database used. This is an ecological study, and the estimates were not adjusted for influential factors such as socioeconomic factors. The finding of the difference between sexes is only on hospitalization, and we cannot input mechanism reasons for it.

## Conclusion

This nationally representative analysis of the Brazilian population’s hospitalization records from 2008 to 2022 demonstrates a clear decrease in ARF hospitalizations over time. Additionally, there is no significant difference in ARF cases between men and women. The study highlights a correlation between socioeconomic factors and disease burden, with low-income groups experiencing a higher rate of ARF hospitalizations.
